# RETRACTED: Melendez et al. Experimental and DFT Study of the Photoluminescent Green Emission Band of Halogenated (−F, −Cl, and −Br) Imines. *Molecules* 2019, *24*, 3304

**DOI:** 10.3390/molecules31091536

**Published:** 2026-05-06

**Authors:** Francisco J. Melendez, María Eugenia Castro, Oscar Portillo-Moreno, Guadalupe Hernández-Téllez, Gloria E. Moreno-Morales, Daniela Gutiérrez-Argüelles, Rodolfo Palomino-Merino, Efraín Rubio-Rosas, René Gutiérrez-Pérez

**Affiliations:** 1 Laboratorio de Química Teórica, Centro de Investigación, Dpto. de Fisicoquímica, Facultad de Ciencias Químicas, Universidad Autónoma de Puebla, Edif. FCQ10, San Claudio y 22 Sur, Ciudad Universitaria, Col. San Manuel, Puebla, Puebla 72570, Mexico; 2 Centro de Química, Instituto de Ciencias, Universidad Autónoma de Puebla, Complejo de Ciencias, ICUAP, Edif. IC8, 22 Sur y San Claudio, Ciudad Universitaria, Puebla 72570, Mexico; mareug.castro@correo.buap.mx; 3 Laboratorio de Síntesis de Complejos. Fac. Ciencias. Químicas, Universidad Autónoma de Puebla, P.O. Box 156, C.P. Puebla Pue 72001, Mexico; osporti@yahoo.com.mx (O.P.-M.); guadalupe.hernandez@correo.buap.mx (G.H.-T.); gloriae_moreno@yahoo.com.mx (G.E.M.-M.); danita_13@hotmail.com (D.G.-A.); jrgutie@correo.buap.mx (R.G.-P.); 4 Posgrado en Optoelectrónica, Facultad de Ciencias Físico-Matemáticas, Universidad Autónoma de Puebla, Pue., P.O. Box 1067, C.P. Puebla Pue 72001, Mexico; palomino@fcfm.buap.mx; 5 Centro Universitario de Vinculación y Transferencia Tecnológica, Universidad Autónoma de Puebla, C.U., C.P. Puebla Pue 72001, Mexico; efrain.rubio@correo.buap.mx

The journal retracts the article titled “Experimental and DFT Study of the Photoluminescent Green Emission Band of Halogenated (−F, −Cl, and −Br) Imines” [1], cited above.

Following publication, concerns were brought to the attention of the Editorial Office regarding image concerns and the scientific accuracy of data presented in this publication [1].

In accordance with our standard procedures, the Editorial Office and the Editorial Board conducted an investigation that identified duplications between Figures 6, 7, 10 and 11 in [1] and multiple figures presented an earlier publication [2], produced by a similar authorship group, presenting different experimental conditions. Although the authors cooperated with the investigation, the explanations and supporting materials provided were not deemed sufficient by the Editorial Board to resolve the identified concerns. Consequently, the Editorial Board has lost confidence in the reliability of the reported findings and has decided to retract this publication.

This retraction was approved by the Editor-in-Chief of the *Molecules* journal.

The authors have been informed of this retraction but did not provide a comment on this decision.
